# Editorial: Neurophysiology in Alzheimer's disease and dementia, volume II

**DOI:** 10.3389/fnagi.2024.1386093

**Published:** 2024-03-07

**Authors:** Davide Vito Moretti

**Affiliations:** Alzheimer's Rehabilitation Unit, IRCCS Istituto Centro San Giovanni Di Dio Fatebenefratelli, Brescia, Italy

**Keywords:** EEG, MEG, fMRI, TMS, rTMS tACS, neurophysiology

Alzheimer's disease (AD) is a progressive neurodegenerative disorder characterized by memory loss and cognitive decline. There is emerging evidence that the pathogenesis of neurodegeneration is related to widespread and progressive changes in brain networking. This can be defined both in structural terms, as patterns of focal and tract neural degeneration, and in functional terms, as altered patterns of brain connectivity and neural transmission.

The gap between what imaging can discern and underlying pathophysiology can now be addressed by advanced techniques that explore the cortical neural synchronization, excitability and functional connectivity that underpin cognitive, motor, sensory and other functions.

A large body of research explores the neurophysiological changes in AD patients over the disease timecourse. Electroencephalography (EEG) and magnetoencephalography (MEG) recordings probe (temporal) synchronization of cortical neuronal activity using sensors placed on (for EEG) or at small distance from (for MEG) the scalp. While the exact mechanisms of the cortical signal generation remains to be understood, there is evidence that scalp-recorded EEG/MEG signals reflect the spatial summation of relatively long-lasting (ten to hundreds of milliseconds) excitatory/inhibitory post-synaptic potentials and dendritic influences of neurons (e.g., the cortical pyramidal neurons), summed together in adjacent regions. In addition to these post-synaptic potentials the EEG/MEG oscillations also originate from the flow of the activity volleys in the longer-range pathways such as thalamo-cortical connections and loops. AD patients show brain oscillatory changes on EEG/MEG compared to healthy older adults.

Functional magnetic resonance imaging (fMRI) has also furthered understanding of AD neurophysiology. fMRI detects changes in blood flow related to neural activity. fMRI reveals AD patients have impaired connectivity in memory-related neural networks. The posterior cingulate cortex, precuneus, and hippocampus show disrupted activation early in disease progression. This research seeks biomarkers to better diagnose and monitor progression of AD neurophysiological dysfunction and aid in developing new treatments. Emerging research has also examined using non-invasive brain stimulation techniques to modulate neurophysiological activity in AD. Transcranial magnetic stimulation (TMS) uses magnetic fields to electrically stimulate neurons. Studies find TMS can temporarily improve memory and cognition in AD patients. TMS may enhance synaptic plasticity and neuronal connectivity. Transcranial alternating current stimulation (tACS) is another approach using mild electrical currents to modulate brain rhythms. Early tACS studies show modulating signaling patterns can affect memory performance. Both TMS and tACS are being explored as potential therapies that may slow cognitive decline in AD by directly altering neurophysiological functioning. Ongoing work continues to refine stimulation parameters and further investigate the feasibility of brain stimulation for AD treatment. These stimulation methods provide additional ways to both study and potentially improve the disturbed neurophysiology seen in Alzheimer's disease.

This Research Topic collects groundbreaking articles that offer novel insights and address the comprehensive issue of the significance of neurophysiology in evaluating normal aging and dementia and with possible therapeutic options.

Adebisi et al. made a systematic review on brain network analysis for dementia disorders using electrophysiology signals aiming to identify methodological issues involved in connectivity analysis frameworks and suggesting approaches to solve such issues. The review is based on the analysis of 41 peer-reviewed articles published between January 2016 and December 2021 and highlights an increase in research in this field during the considered period. The authors observe that most articles use graph theory metrics for the analysis, such as clustering coefficient, characteristic path length, and global efficiency. This review has practical implications for the diagnosis and treatment of dementia-related disorders, as it provides insight into how to employ connectivity measures for the analysis of electrophysiology signals, thus contributing to a better understanding of their underlying mechanisms. The systematic review found that the ratio of MEG and EEG utilization in the reviewed articles was 1:9. Additionally, most of the reviewed articles employed graph theory metrics for their analysis, with clustering coefficient (CC), characteristic path length (CPL), and global efficiency (GE) being the most frequently used metrics. Based on the reviewed articles, the suggested approaches for improving connectivity analysis frameworks for dementia disorders using electrophysiology signals include identifying methodological issues involved in such frameworks and proposing approaches to solve them. The study also highlighted an increasing trend of research in this domain and revealed that most of the reviewed articles employed graph theory metrics for their analysis, with clustering coefficient (CC), characteristic path length (CPL), and global efficiency (GE) being the most frequently used metrics.

Ponomareva et al.'s study investigated the neurophysiological alterations in individuals with preclinical and early manifest Huntington's disease (HD) using EEG and fMRI. The findings showed a decrease in power in specific frequency ranges in the EEG of preHD individuals, as well as a reduction in power within the classic alpha band in EMHD patients. Disrupted functional connectivity was observed in various brain networks, particularly within the frontal lobe, putamen-cortical, and cortico-cerebellar networks, in individuals with the HD mutation. The analysis also revealed an association between decreased alpha power and increased connectivity in large-scale brain networks. These findings suggest that EEG and fMRI can provide valuable information for monitoring the progression of HD. The neurophysiological alterations observed in individuals with preclinical Huntington's disease (HD) included a decrease in power in a specific frequency range at the theta-alpha border and slow alpha activity in the EEG. This indicates changes in brain activity even before the onset of clinical symptoms. The study found disrupted functional connectivity in various brain networks, particularly within the frontal lobe, putamen-cortical, and cortico-cerebellar networks in individuals with the HD mutation. The significance of the association between decreased alpha power and increased connectivity in large-scale brain networks is that it suggests a relationship between neurophysiological changes in individuals with early manifest Huntington's disease (EMHD) and alterations in brain network connectivity. This association may provide valuable insights into the pathological processes underlying the development of Huntington's disease and could potentially contribute to the monitoring and understanding of disease progression.

Chen et al.' study investigated the changes in cross-frequency coupling (CFC) properties in patients with mild cognitive impairment (MCI) and Alzheimer's disease (AD) using resting-state EEG signals. The findings suggest that AD and MCI patients exhibit enhanced CFC between low-frequency and high-frequency oscillations, with MCI patients also showing enhanced delta-gamma and theta-gamma couplings in specific brain regions. These CFC properties were found to correlate with cognitive function, particularly in the memory domain. The study measured cross-frequency coupling (CFC) in the EEG signals of MCI and AD patients by recording resting-state EEG (rsEEG) signals in 46 MCI patients, 43 AD patients, and 43 cognitively healthy controls (HCs). The researchers then analyzed the changes in CFC and the relationship between CFC and scores on clinical tests of cognitive function. The specific cognitive domains found to be correlated with the observed changes in cross-frequency coupling (CFC) properties were memory function domain. These findings suggest that alterations in CFC properties are significantly correlated with various cognitive domains, especially in the memory function domain. The findings of the study have significant implications for the diagnosis and understanding of Alzheimer's disease (AD) and mild cognitive impairment (MCI). The study revealed that AD and MCI patients exhibit enhanced cross-frequency coupling (CFC) between different frequency bands in their EEG signals. Specifically, multiple couplings between low-frequency oscillations and high-frequency oscillations were found to be significantly enhanced in AD patients compared to healthy controls and MCI patients. Moreover, delta-gamma and theta-gamma couplings in specific brain regions were significantly enhanced in MCI patients compared to healthy controls. These CFC properties were found to correlate significantly with various cognitive domains, especially the memory function domain. Therefore, these findings suggest that alterations in CFC properties could serve as valuable indicators for the progression of these disorders and may provide insights into the neural mechanisms underlying cognitive impairment in AD and MCI.

Lanza et al. made a comprehensive review of transcranial magnetic stimulation (TMS) in secondary dementia. It emphasizes the need for diagnostic tools to identify and monitor treatable cognitive disorders and predicts the response to treatment. The review discusses various conditions that can cause secondary dementia, such as normal-pressure hydrocephalus, multiple sclerosis, celiac disease, and other immunologically-mediated diseases, as well as inflammatory, infective, metabolic, toxic, nutritional, endocrine, sleep-related, and rare genetic disorders. It also highlights the importance of understanding the pathological mechanisms of cognitive impairment in secondary dementia and the potential for TMS-associated measures of cortical function and plasticity to provide useful information in combination with clinical features and other diagnostic tests. The authors suggest that measures of cortical function and plasticity associated with TMS, such as the short-latency afferent inhibition, the short-interval intracortical inhibition, and the cortical silent period, could be useful in evaluating secondary dementias, especially when combined with clinical features and other diagnostic tests. Finally, the review highlights the lack of a comprehensive review of TMS studies available in other secondary dementias, such as normal-pressure hydrocephalus, multiple sclerosis, celiac disease, and other immunologically-mediated diseases, as well as a number of inflammatory, infective, metabolic, toxic, nutritional, endocrine, sleep-related, and rare genetic disorders. It emphasizes the need for further understanding of the pathological mechanisms of cognitive impairment in secondary dementia and the potential for TMS-associated measures of cortical function and plasticity to fill the gap in the literature.

Saitoh et al. in a randomized, sham-controlled clinical trial conducted in Japan, investigated the efficacy and safety of repetitive transcranial magnetic stimulation (rTMS) were evaluated in patients with Alzheimer's dementia. Forty-two patients aged 60–93 years were enrolled and received either active rTMS or a sham treatment. The results showed that there were no significant differences in cognitive and functional scores between the groups. However, a *post-hoc* analysis revealed that participants with higher baseline cognitive scores showed significant improvement in the active rTMS group compared to the sham group. The improvement was not sustained at week 20. No serious adverse events related to the intervention were reported. The outcomes of the randomized, sham-controlled clinical trial evaluating the efficacy of repetitive transcranial magnetic stimulation (rTMS) in patients with Alzheimer's dementia showed that there were no significant differences in cognitive and functional scores between the active rTMS and sham treatment groups. However, a *post-hoc* analysis revealed that participants with higher baseline cognitive scores showed significant improvement in the active rTMS group compared to the sham group. The improvement was not sustained at week 20, and no serious adverse events related to the intervention were reported. Based on the randomized, sham-controlled clinical trial evaluating the efficacy of repetitive transcranial magnetic stimulation (rTMS) in patients with Alzheimer's dementia, the outcomes showed no significant differences in cognitive and functional scores between the active rTMS and sham treatment groups. However, a *post-hoc* analysis revealed that participants with higher baseline cognitive scores showed significant improvement in the active rTMS group compared to the sham group. The improvement observed in the active rTMS group was not sustained over time. A *post-hoc* analysis revealed that participants with higher baseline cognitive scores showed significant improvement in the active rTMS group compared to the sham group. However, the efficacy disappeared by week 20, based on the Alzheimer's Disease Assessment Scale-Cognitive (ADAS-cog) and Montreal Cognitive Assessment (MoCA-J) scores.

Zhang et al. in their systematic review and meta-analysis evaluated the effects of repetitive transcranial magnetic stimulation (rTMS) combined with cognitive training on cognitive function in patients with Alzheimer's Disease (AD). The analysis included ten studies with a total of 408 participants. The results showed that the addition of rTMS significantly improved overall cognition in AD patients compared to cognitive intervention alone. The treatment also had some continuity, with significant improvements in cognitive function persisting even after the treatment ended. The study suggests that rTMS combined with cognitive training is a valuable technique for the cognitive rehabilitation of AD patients. Repetitive transcranial magnetic stimulation (rTMS) is a non-invasive brain stimulation technique that uses a pulsating magnetic field to induce electrical currents in specific areas of the brain. This can result in the modulation of neuronal activity, which may lead to improvements in cognitive function. In the context of Alzheimer's Disease (AD) patients, rTMS paired with cognitive training has been shown to significantly improve overall cognition and cognitive function, making it a valuable technique for the cognitive rehabilitation of AD patients. Cognitive training complements the effects of repetitive transcranial magnetic stimulation (rTMS) in improving cognitive function by providing targeted exercises and activities to stimulate and enhance specific cognitive abilities. When combined with rTMS, cognitive training can help reinforce and consolidate the improvements in neuronal activity induced by rTMS, leading to more significant and sustained enhancements in cognitive function. This combination has been shown to be beneficial in improving the cognitive ability of Alzheimer's Disease (AD) patients and restoring their overall functional state.

Liu G. et al. conducted a randomized, sham-controlled, clinical trial was conducted to evaluate the treatment outcomes of repetitive transcranial magnetic stimulation (rTMS) in patients with moderate-to-severe Alzheimer's disease (AD). The study included 35 AD patients who underwent a 3-month treatment procedure of high-frequency rTMS stimulation on the left dorsal lateral prefrontal cortex. The participants completed neuropsychological tests before and after the treatment, and 12 of them also underwent resting-state functional magnetic resonance imaging (fMRI) to explore the neural contribution to treatment outcomes. The results showed that rTMS treatment significantly improved cognitive performance, reduced psychiatric symptoms, and improved the clinician's global impression of change. Additionally, the study proposed a pre-treatment neuroimaging marker in the (para)hippocampal region and frontal and occipital cortices for predicting individual differences in treatment outcomes. The specific neuropsychological tests used to evaluate the treatment outcomes of repetitive transcranial magnetic stimulation (rTMS) in patients with moderate-to-severe Alzheimer's disease included the severe impairment battery (SIB) and the neuropsychiatric inventory (NPI). These tests were completed by the participants at baseline and post-treatment to assess the therapeutic effect of rTMS on cognitive performance and psychiatric symptoms. The sham-controlled aspect of the clinical trial was implemented by using a randomized, sham-controlled design. This means that participants were randomly assigned to either the active rTMS treatment group or the sham treatment group, where the sham treatment mimicked the sensory experience of rTMS without delivering the actual magnetic stimulation. This design allowed for a comparison between the effects of the active rTMS treatment and the sham treatment to evaluate the true therapeutic effect of rTMS in patients with moderate-to-severe Alzheimer's disease. The proposed neuroimaging marker for predicting treatment outcomes in patients with moderate-to-severe Alzheimer's disease involves resting-state multivariate functional connectivity in the (para)hippocampal region as well as two clusters in the frontal and occipital cortices. This marker was preliminarily proposed based on the findings of a study that used resting-state functional magnetic resonance imaging (fMRI) to explore the underlying neural contribution to individual differences in treatment outcomes. The study suggested that these neuroimaging markers could serve as pre-treatment indicators for predicting individual differences in the response to repetitive transcranial magnetic stimulation (rTMS) therapy.

Liu Y. et al. discuss a study protocol for a randomized controlled trial that aims to evaluate the safety and effectiveness of combining transcranial alternating current stimulation (tACS) with sound stimulation in improving cognitive function in patients with Alzheimer's disease (AD). The trial plans to recruit 87 patients with mild to moderate AD and randomly divide them into three groups. The main evaluation index will be the change in Alzheimer's Disease Assessment Scale-Cognitive (ADAS-Cog) score from before treatment to the end of treatment and 4 months after treatment. The study also aims to explore the changes in brain networks and metabolism in each group after treatment. Transcranial alternating current stimulation (tACS) is a non-invasive brain stimulation technique that involves applying a low-intensity electrical current to the scalp to modulate brain activity. The electrical current oscillates at a specific frequency and is thought to entrain neural oscillations in the brain, influencing cognitive processes. Sound stimulation is combined with transcranial alternating current stimulation (tACS) to improve the long-term post-effect of tACS. This combination aims to maintain the long-term cognitive improvement in patients with Alzheimer's disease. The study protocol involves exploring the safety and effectiveness of tACS combined with sound stimulation and its impact on the cognition of Alzheimer's disease patients. The main evaluation index will be the change in Alzheimer's Disease Assessment Scale-Cognitive (ADAS-Cog) score from before treatment to the end of treatment and 4 months after treatment. The study also aims to explore the changes in brain networks and metabolism in each group after treatment. The potential implications of the study for the treatment of Alzheimer's disease include the exploration of a new, non-invasive joint intervention to improve patients' cognitive status. The study aims to conclude that transcranial alternating current stimulation (tACS) combined with sound stimulation is safe and tolerable in patients with mild to moderate Alzheimer's disease under three standardized treatment regimens. The combination of tACS with sound stimulation is expected to have a significant long-term effect on cognitive improvement compared to tACS alone or sound alone, potentially providing a better treatment plan for Alzheimer's disease patients.

This Research Topic add another step toward understanding the complex relationship between brain networks function and AD pathology. It is an important contribution to pave the way for future studies on AD investigating the relationship between the onset of clinical symptomatology with specific neurophysiological biomarkers both diagnostic and prognostic. The assessment of low cost biomarkers, such as EEG, during screening, may help to identify subjects at risk to develop dementia. Moreover, the longitudinal investigation of the temporal course of progression in at risk individuals could open a new avenue for both disease prevention and the development of new therapeutic strategies.

## Author contributions

DM: Writing—original draft, Writing—review & editing.

